# Occupational hazards and early retirement: German National Cohort (NAKO) baseline distributions

**DOI:** 10.1093/eurpub/ckac130.132

**Published:** 2022-10-25

**Authors:** J Hegewald, K Romero Starke, U Latza, A Seidler

**Affiliations:** 1 Unit 3.1 Prevention of Work-related Diseases, Federal Institute for Occupational Safety and Health, Berlin, Germany; 2 IPAS, TU Dresden, Dresden, Germany; 3 Division 3 Work and Health, Federal Institute for Occupational Safety and Health, Berlin, Germany

## Abstract

**Background:**

Despite measures to ensure occupational safety, harmful workplace conditions that negatively impact worker health persist. The baseline assessment of the German National Cohort (NAKO) provides a basis for determining the prevalence of occupational hazards and early retirement (due to health reasons).

**Methods:**

From 2014 to 2017, the NAKO examined 205,141 participants aged 20-69 years at 18 study centers across Germany. Working participants were asked about exposure to airborne particulates, occupational noise, evening and night work, sick days, presenteeism, and personal safety equipment use (respiratory masks, hearing protection). The assessment also included questions on retirement. We examined the distributions descriptively.

**Results:**

A total of 84.2% (n = 172,766) participants were ever employed. Of these participants, 7.9% reported ever working in a job with dust or air particulate exposure where a respiratory mask was required for at least one year. However, nearly one-third (31.6%) of the particulate-exposed workers reported not having worn a mask. 11.4% worked at least a year in a job that required hearing protection, but 27.2% of noise-exposed workers did not comply with this safety measure. Over half of the workers sometimes worked between 6 and 10 pm, and 18.7% sometimes worked at night (11 pm to 6 am) in the last three months. On average, participants used 10.5 sick days in the previous year, and the 12-month prevalence of going to work sick at least one day (presenteeism) was 64.9%. Nearly one-fourth (24.4%; n = 9043) of retirees reported early retirement due to health reasons.

**Conclusions:**

Hazardous working conditions were common, and there was only partial compliance with the use of available personal safety equipment and measures. The NAKO provides a basis for examining the distribution of occupational exposures in Germany, and future prospective data will permit an evaluation of the effectiveness of preventative regulations.

**Key messages:**

- The NAKO cohort provides essential information on the prevalence of hazardous working conditions in Germany.
- In the future, trend observations from the prospective data can help evaluate the efficacy of preventive regulations.

